# Beta-Galactosidase Staining in the Nucleus of the Solitary Tract of Fos-Tau-LacZ Mice Is Unaffected by Monosodium Glutamate Taste Stimulation

**DOI:** 10.1371/journal.pone.0107238

**Published:** 2014-09-05

**Authors:** Jennifer M. Stratford, John A. Thompson

**Affiliations:** 1 Rocky Mountain Taste & Smell Center, Department of Cell and Developmental Biology, University of Colorado School of Medicine, Aurora, CO, United States of America; 2 Department of Neurosurgery, University of Colorado School of Medicine, Aurora, CO, United States of America; German Institute for Human Nutrition, Germany

## Abstract

Fos-Tau-LacZ (FTL) transgenic mice are used to visualize the anatomical connectivity of neurons that express c-Fos, an immediate early gene, in response to activation. In contrast to typical c-Fos protein expression, which is localized to the nucleus of stimulated neurons, activation of the c-Fos gene results in beta galactosidase (β-gal) expression throughout the entire cytoplasm of activated cells in FTL mice; thereby making it possible to discern the morphology of c-Fos expressing cells. This can be an especially important tool in brain areas in which function may be related to cell morphology, such as the primary taste/viscerosensory brainstem nucleus of the solitary tract (nTS). Thus, to further characterize FTL activity in the brain, the current study quantified both β-gal enzymatic activity as well as c-Fos protein expression in the nTS under a variety of experimental conditions (no stimulation, no stimulation with prior overnight food and water restriction, monosodium glutamate taste stimulation, and monosodium glutamate taste stimulation with perfusion 5 h post stimulation). Contrary to previous research, we found that β-gal activity (both labeled cell bodies and overall number of labeled pixels) was unchanged across all experimental conditions. However, traditional c-Fos protein activity (both cell bodies and number of activated pixels) varied significantly across experimental conditions, with the greatest amount of c-Fos protein label found in the group that received monosodium glutamate taste stimulation. Interestingly, although many c-Fos positive cells were also β-gal positive in the taste stimulated group, some c-Fos protein labeled cells were not co-labeled with β-gal. Together, these data suggest that β-gal staining within the nTS reflects a stable population of β-gal- positive neurons whose pattern of expression is unaffected by experimental condition.

## Introduction

The immediate early gene, c-Fos, has been used extensively as an anatomical marker of neuronal activity since the early 1980s [1], with over 19,000 publications employing this technique. In particular, c-Fos protein immunohistochemistry is invaluable for quantifying the spatial distribution of neuronal activity within the brain. Use of c-Fos as a neuronal activity marker enables visualization of spatial patterns of brain activation throughout a region of interest as well as the co-localization of secondary and tertiary cellular markers that c-Fos positive cells express (e.g. neuropeptides, enzymes, etc.,), making identification of an activated cell's neuronal phenotype possible. Yet, because c-Fos protein staining is limited to the nucleus of activated cells, determination of the morphology of activated cells remains unknown. This is of particular importance in brain areas whose function may be dependent upon both regional localization and cell morphology, such as the nucleus of the solitary tract (nTS; primary taste/viscerosensory nucleus; [2]).

One way to address this concern is to use the transgenic mouse line Fos-tau-LacZ (FTL), which makes it possible to discern the morphology of activated, c-Fos positive cells for the first time. In FTL mice, neuronal activation results in expression of the β - galactosidase enzyme (β-gal; via the LacZ gene). Coupling of the LacZ gene with a tau reporter results in shuttling of β-gal throughout the cytoplasm of cells, thereby thoroughly labeling the cellular architecture (e.g. dendrites and axon) of activated cells [3]. Because the β-gal enzyme is first synthesized prior to transportation throughout the cytoplasm of activated cells, this results in a delay in β-gal activity compared to c-Fos protein (β-gal expression peaks +4 hours post stimulation, whereas c-Fos protein expression peaks 1–3 hours following stimulation; [4], [5]).

Though this altered time course was previously characterized in other brain regions, such as the hippocampus, little is known about β-gal activity in brainstem regions, including the nTS. Given the multimodal nature of the nTS, which serves as an integration hub for gustatory, thermal, mechanosensory, viscerosensory, and cardiovascular information, it is likely that β-gal activity in the nTS differs from other brain areas. Thus, the present study sought to quantify β-gal staining in the nTS under different experimental conditions.

## Methods

### Animals

Adult Fos-Tau-LacZ (FTL) male and female mice (M = 7, F = 5; age 8–11 months) were used. Animals were genotyped by PCR using the same primers and protocol utilized previously [3]. One additional wildtype (WT) and one additional FTL mouse (both female, 10 and 11 months old, respectively) were used to compare stimulated c-Fos protein activity between WT and FTL mice. The animals were housed in a vivarium with a 12 h light/dark cycle with lights on at 0500. Food (Teklad Global Rodent Diet # 2918) and water were available ad libitum throughout the course of the experiment, except as noted.

All animal procedures were performed in accordance with NIH guidelines and were approved by the Institutional Animal Care and Use Committee at the University of Colorado Denver School of Medicine.

### Stimulation procedures

In order to thoroughly characterize c-Fos protein and β-gal activity in the nTS, animals were placed into one of four stimulation groups (n = 3 in each group). Baseline c-Fos/β-gal was determined by comparing the amount of c-Fos/β-gal expression in animals that either received no stimulation (Unstim) or no stimulation after being deprived of food and water overnight (18 hours; No Food/Water).

To characterize the time course of c-Fos protein and β-gal activity after taste stimulation, animals were allowed to freely ingest monosodium glutamate (MSG) from a drinking bottle and then were euthanized and perfused at two different times following stimulation (MSG and MSG 5 h Post, see below for details). To do this, three days prior to the experiment, mice were placed on 23 hour/day water restriction. During this time, animals were given 1 hour access to water in a single drinking bottle at the same time each day to train animals to consume fluids in a relatively short period of time. On stimulation day, animals were given 150 mM MSG contained in a single drinking bottle in their home cage for 30 minutes. At the end of 30 minutes, fluid intake was recorded and animals were left undisturbed for either 45 min (MSG) or 5 hours (MSG 5 h Post) prior to perfusion (see *Immunohistochemistry*). During MSG stimulation, fluid intake was consistent across all animals (Mean ± S.D.: 2.9±0.3 ml).

### Immunohistochemistry

Following MSG taste stimulation, animals were deeply anesthetized with Fatal-Plus (50 mg/kg intraperitoneally; MWI, Boise, ID) and perfused transcardially first with 0.9% saline and then with 4% paraformaldehyde in 0.1 M phosphate buffer. After perfusion, the brain was removed and post-fixed in 4% paraformaldehyde for 3–5 hours. Then tissue was placed in 20% sucrose overnight at 4°C. The olfactory bulbs (control) and brainstem were isolated, embedded in Optimal Cutting Temperature compound (Fisher Scientific, Pittsburgh, PA) and frozen rapidly on dry ice. Coronal sections (40 µM) were cut on a cryostat and divided into a series of three adjacent sets. Sections were either reacted immediately or placed in cryoprotectant and stored at −20°C [6] for later processing. Sections stored in cryoprotectant were thoroughly rinsed in 0.1 M phosphate- buffered saline (PBS) before staining.

#### Primary and Secondary antibody incubations

All steps were conducted at room temperature unless otherwise indicated. Sections were first washed in 0.1M PBS. Sections were then blocked in 2% normal donkey serum (NDS, Jackson ImmunoResearch, West Grove, PA) for 1 hour and then incubated with both rabbit anti-Fos and guinea pig anti- β-gal diluted in antibody media (AB media; 0.3% triton, 0.15 M sodium chloride, and 1% bovine serum albumin; for details about anti-sera see below) at 4°C for 48 hours (c-Fos) or 24 hours (β-gal).

Following primary antibody incubation and three washes in 0.1 M PBS, the anti-Fos antibody was detected with a Rhodamine Red-X fab fragment donkey anti-rabbit antibody and the anti- β-gal antibody was detected with an Alexa Fluor 488 donkey anti-guinea pig antibody (both Jackson ImmunoResearch Laboratories catalog #s 711-297-003 and 706-545-148; Lot #s103577 and 105625, respectively; each diluted 1∶800), which were incubated with tissue for two hours. All tissue was also counterstained with NeuroTrace 640/680 1∶500 (Nissl stain, lot # 927003, Invitrogen, Carlsbad, CA), which was included in the antibody media during secondary antibody incubation. Following an additional three 0.1M PBS washes, free floating tissue sections were mounted onto Superfrost Plus slides (Fisher Scientific, Pittsburgh, PA), and then cover slipped using Fluoromount – G (Southern Biotech, Birmingham, AL).

#### c-Fos and β-gal antisera

Sections were processed for c-Fos- like immunoreactivity (Fos-LI) using a rabbit polyclonal anti-c-Fos antibody from Millipore (formerly sold by Calbiochem and Oncogene; RRID AB_213663). The c-Fos antibody (Ab-5, catalog # PC38; lot # D00134698; diluted 1∶3,000) was prepared against a peptide mapping at residues 4–17 of the human c-Fos protein. This antiserum stains a single band at ∼50–55 kDa as observed by Western Blot analysis of Fibroblast-like BHK 21 C13 cells [7]. In the current study, omission of rabbit anti-c-Fos primary antibody resulted in no labeled cells (data not shown).

β-gal staining was visualized using a guinea pig polyclonal anti β-gal antibody custom made by Antibodies Incorporated (Davis, CA). The β-gal antibody (diluted 1∶1000) was prepared against the entire β-Galactosidase sequence by inoculating a guinea pig with the galactosidase G5635 immunogen from Sigma (Saint Louis, MO). No β-gal staining was observed in tissue from LacZ - negative FTL littermates, nor in LacZ -positive FTL animals when the β-gal primary antibody was omitted (data not shown).

### Microscopic analysis

Image acquisition exposure times for each fluorophore were the same across all animals. Whole slide, fluorescent images were photographed using Surveyor by Objective Imaging (Cambridge, UK), software that controls the microscope stage as well as enables image acquisition, with a black and white Leica DFC 365FX camera on a Leica DM6000B microscope. To capture whole-slide scans, the ‘Multiscan’ option in the imaging software was used. For each fluorophore, an overlapping grid of images was captured using a 10× objective. Within each grid, all three channels (FITC, Texas Red, Cy-5) were obtained sequentially and merged together to prevent side-band excitation of the different fluorophores. Images were then stitched together in real time using the ‘Best Focus’ algorithm in the Surveyor software, which yielded a mosaic image of the whole microscope slide. Images of individual fluorescent, RBG brain sections were then obtained using the ‘Region of Interest Tool’ in the Surveyor Viewer Software (Cambridge, UK; see Figures S1, S2, S3, S4, S5, S6, S7, S8, S9, S10, S11, S12, S13, S14, S15, S16, S17, S18, S19, S20, S21, S22, S23, S24, S25, S26, S27, S28, S29, S30, S31, S32, S33, S34, S35, S36 for raw images).

### Cell count and pixel number quantification

To characterize c-Fos and β-gal activity throughout the rostral-caudal extent of the nTS, staining was visualized and quantified in three representative levels: Rostral, Intermediate, and Caudal (situated respectively at −6.72, −7.08, and −7.56 from bregma; or −0.36, −0.72, −1.20 mm relative to the rostral-most (i.e. ‘r1’) portion of the nTS as defined previously [17]). Moreover, the Rostral, Intermediate and Caudal nTS levels in the present study correspond to the ‘r3’ ‘i1,’ and ‘i5 nTS levels previously described [17]. To quantify the number of c-Fos positive and β-gal positive cells, the red (c-Fos) and green (β-gal) color channels in each image were first filtered with a stringent threshold (mean +2 standard deviations of background pixel intensity level), and each color channel was then converted to a binary, black/white (BW) image using *ImageBWconvertGUI*- a custom-made program running in the 2013a Matlab Computing Environment with the Image Processing Toolbox (The MathWorks, Natick, MA; program available on Github: https://github.com/neuropil/ImageBWconvert/). This resulted in binary BW images representing c-Fos and β-gal staining >2 standard deviations of background staining for each animal. Then, the number of c-Fos positive and β-gal- positive cell bodies was quantified using the cell counter plugin in ImageJ (version 1.47, Bethesda, MD). The number of c-Fos/β-gal double labeled cells was quantified by first converting each filtered binary B & W image to magenta (c-Fos) or green (β-gal) layers in Photoshop (Adobe Design Standard CS5, San Jose, CA) using the Channels and Layers tools. The magenta and green layers were overlaid using the ‘Lighten’ option in the Layers drop down menu. Then the number of double labeled cells was quantified using the cell counter plugin in ImageJ. Cases were counted only when substantial Fos-LI was observed in the olfactory bulb, as c-Fos expression is robust in the olfactory bulb in all animals [8] (see Figure S37 for a representative photomicrograph of c-Fos/β-gal staining in the olfactory bulb).

In addition to quantification of the number of c-Fos and β-gal positive cell bodies, we also quantified, across different experimental conditions, the number and percentage of pixels within each nTS polygon with a pixel intensity value that exceeded 2 standard deviations for each stain, which includes both histological-positive cell bodies as well as associated cellular morphology (e.g., dendrites and axons) using *ROIImageAnalysis* - another custom-made MATLAB program (available on Github: https://github.com/neuropil/ROIImageAnalysis/).

To obtain an unbiased measure of c-Fos and β-gal staining throughout the nTS, the total number of pixels for each label that exceeded a pixel intensity threshold (mean +2 standard deviations), derived from within the boundary of nTS, was measured. To do this, the threshold was calculated by taking the mean and standard deviation of the aggregate of pixel intensity values from all nTS regions (extracted through region-of-interest polygons traced in *ROIImageAnalysis*) across all sections for all experimental groups. Obtaining the pixel intensity threshold from all sections normalizes the variability associated with brain preparation and immunohistochemistry. To calculate the percentage of pixels exceeding threshold for each label, we divided the number pixels above threshold (i.e., area of expression) by the total number of pixels in the traced polygon (i.e., area of nTS section).

Measuring the total pixel number exceeding threshold and calculating the percentage of nTS with positive expression are both important tools because the total labeled pixel number provides an indirect quantification of expression across elements of cellular architecture (soma, dendrites and axon); whereas the percentage of labeled nTS provides quantification of expression density. After deriving the threshold across all nTS sections, boundaries of the nTS were retraced using only the Nissl image (following conversion to inverted grayscale: label = black and no-label = white). This method of boundary extrapolation was used to ensure that manual tracing of nTS boundaries was not biased by inclusion of adjacent label (i.e. only viewing the Nissl stain). The *ROIImageAnalysis* program then extracted the x-y coordinates of the polygon traced in the Nissl channel and applied them to the other label channels (e.g. Red and Green) to extract and calculate the number of pixels within the polygon that exceeded threshold as well as the percentage of nTS that contained labeled pixels.

### Statistics

Data are presented as group means ± S.E. Data were analyzed using appropriate two - way (group x nTS level) ANOVAs (Statistica; StatSoft, Tulsa, OK). Tukey's honest significant difference tests were used to assess statistically significant (p<0.05) main effects or interactions.

## Results

### β- galactosidase cell counts

**Figure 1:**
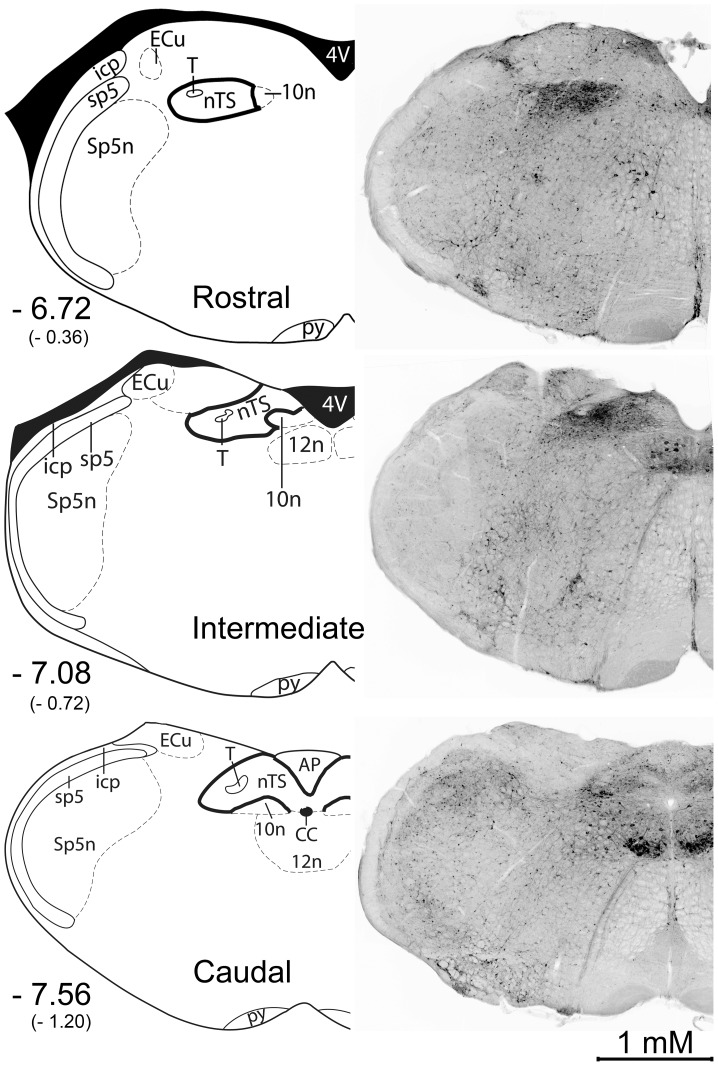
β – galactosidase staining is found throughout the brainstem, but is especially dense within the nTS and hypoglossal nucleus. **Left**: Three rostrocaudal nTS levels designated as Rostral, Intermediate, and Caudal. Coordinates from Bregma are listed for each nTS level. Numbers in parenthesis represent coordinates relative to the rostral-most (i.e. ‘r1’) portion of the nTS as defined previously [17]. AP: area postrema, CC: central canal, ECu: external cuneate nucleus, icp: inferior peduncle, nTS: nucleus of the solitary tract, py: pyramidal tract, sp5: spinal trigeminal tract, Sp5n: spinal trigeminal nucleus, T: solitary tract, 4V: 4th ventricle, 10n: dorsal motor nucleus of the vagus, 12n: hypoglossal nucleus. Images modified from Paxinos The Mouse Brain in Stereotaxic Coordinates, 2nd Edition. **Right**: Photomicrographs of fluorescent β – galactosidase staining in the brainstem of a FTL mouse that received no stimulation (Unstim) Images converted to greyscale colors for clarity.

**Figure 2:**
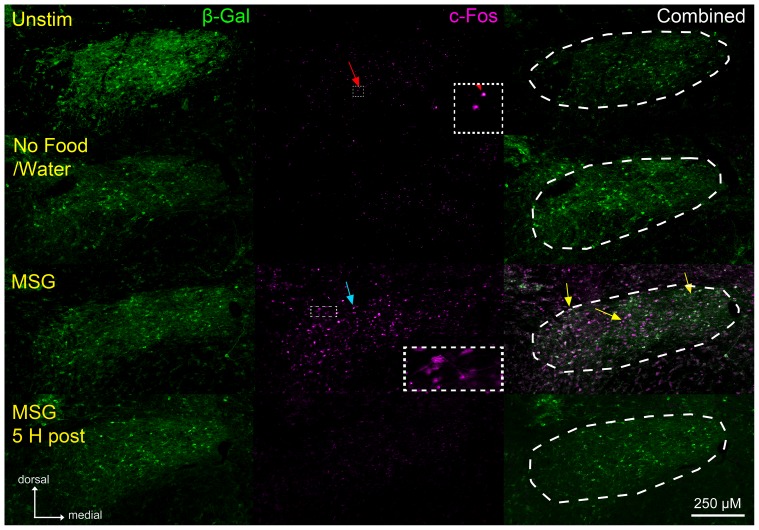
β – galactosidase staining is similar across experimental conditions; whereas c-Fos protein label varies significantly. **Left**: β-gal staining (green) is not different across all experimental groups. **Middle**: Significant c-Fos staining (magenta) is present only in the MSG group. However, punctate c-Fos staining (red arrow) is found in all other groups. Moreover, c-Fos protein staining is found in the cellular processes of some c-Fos labeled cells in the MSG group (inset). **Right**: A significant proportion of doubled labeled c-Fos/β-gal cells (white label and left most yellow arrow) is found in the MSG group. Interestingly, we observed some c-Fos positive cells that were not co-labeled with β-gal (middle yellow arrow) as well as some β-gal positive cells that did not co-localize with the c-Fos protein (right yellow arrow). Sections shown are of the ‘Rostral’ representative level.

**Figure 3:**
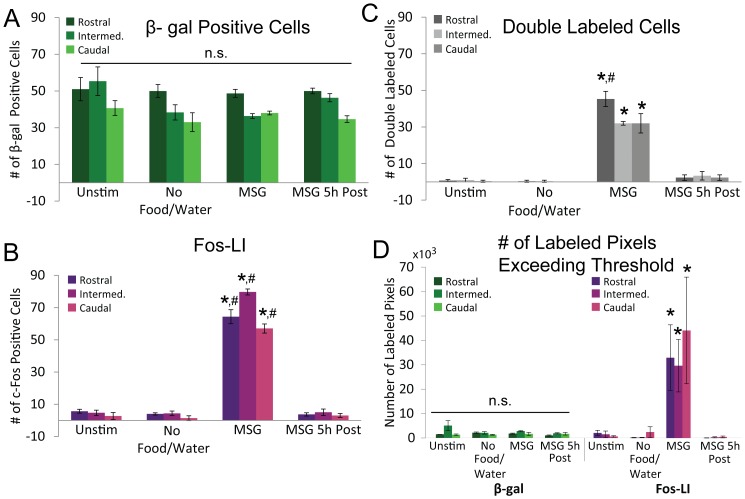
Bar graphs summarizing all quantative measurements across experimental conditions. **A**: The number of β-gal positive cells (green) was not different within each nTS level (Rostral, Intermediate, Caudal), nor between stimulation groups (Unstim, No Food/Water, MSG, MSG 5 h Post, p = 0.25). Interestingly, the number of β-gal positive cells, irrespective of group, was significantly different between nTS levels (Rostral>Intermediate>Caudal; all p's>0.05, data not shown). **B**: In contrast to the number of β-gal positive cells, the number of c-Fos positive cells (Fos-LI; magenta) was significantly different between groups, with Fos-LI in the MSG group greater than all other groups. Also, within the nTS of the MSG group, Fos-LI was significantly different between nTS levels (Intermediate (Intermed.)>Rostral>Caudal). **C**: The number of c-Fos/β-gal double labeled cells matched the Fos-LI results seen in *C*, with the number of double labeled cells significantly greater in the MSG group as compared to all other groups. Also, the number of double labeled cells was significantly greater in the Rostral level of the nTS in the MSG group compared to both the Intermed. and Caudal groups. **D**: The number of β-gal (green) labeled pixels that exceeded threshold (which includes both cell bodies and cellular processes) was not statistically different between groups (p = 0.36). However, the number of c-Fos labeled pixels (magenta) was significantly greater in the MSG group as compared to all other groups. In all figures: n.s. = not statistically significantly different; * = a significant difference between the MSG group vs. all other groups; # = a significant different between nTS levels within the MSG group. All p's<0.05.

Robust β-gal staining was found throughout the entire brainstem of animals that received no stimulation (Unstim), with a particularly dense concentration in the nTS (Figure 1). Unfortunately, as seen in Figure 1, this dense mesh of β-gal staining made determination of the morphology of individual activated cells within the nTS impossible. Interestingly, strong β-gal staining was also observed in the olfactory blub of FTL mice (Figure S37). Moreover, the number of β-gal positive cells did not change across all experimental conditions ([F (6, 16) = 1.47, p = 0.25]; Figure 2, left column; Figure 3A). Furthermore, although the number of β-gal positive cells was not different between groups, the overall number of β-gal positive cells was significantly different between nTS levels (Rostral, 59.9±1.6, >Intermediate, 44.1±3.0, >Caudal, 36.6±1.7; all p's<0.05).

### Fos-LI cell counts

In contrast to β-gal staining, the number of c-Fos positive cells varied significantly across conditions ([F (6, 16) = 20.91, p<0.05]). A low quantity of Fos-LI was observed in the nTS of animals that received either no stimulation (Unstim) or no stimulation following overnight food and water restriction (No Food/Water); whereas, significant Fos-LI was seen in the nTS of animals that consumed 150 mM MSG (MSG), but not in the nTS of animals that were allowed to drink MSG and were perfused 5 h later (MSG 5 h Post; Figure 2, middle column; Figure 3B). Further, Fos-LI in the nTS of MSG animals was significantly greater than any other group at all three levels (Rostral, Intermediate, Caudal; all p's<0.05).

In addition to labeled cell bodies, three unusual Fos-LI staining patterns were observed. First, basal punctate Fos-LI was seen in both unstimulated groups (Unstim and No Food/Water) as well as in the MSG 5 h Post group (Figure 2, middle column, red arrow). However, this staining did not resemble traditional c-Fos protein nuclear staining (Figure 2, middle column, blue arrow) and was not the result of non-specific secondary antibody binding as this staining was not present when the primary antibody was omitted (data not shown). Moreover, usually high background staining, defined as non-specific immunofluorescence that was not localized to cell nuclei, was found within the nTS of MSG-stimulated FTL animals (Figure 2, middle column), but was not present in the nTS of MSG-stimulated WT control mice (tissue was processed concurrently with that of FTL mice, using the same antibodies and reagents; see Figure S38). Lastly, Fos-LI was found in the cellular processes of some c-Fos positive cells in the nTS of MSG-stimulated animals (Figure 2, middle column, inset).

### c-Fos/β- galactosidase double labeled cells

Similar to the pattern observed with respect to the number of c-Fos positive cells, the number of c-Fos/β-gal double labeled cells was significantly greater in the MSG group as compared to all other groups ([F (6, 16) = 8.49, p<0.05]). Furthermore, the number of double labeled cells was significantly greater in the Rostral nTS level of the MSG group than either the Intermediate or Caudal levels (all p's<0.05; Figure 2, right column and Figure 3C). Although a large portion of c-Fos/β-gal double labeled cells were observed in the MSG group, some c-Fos labeled cells did not co-localize with β-gal and some β-gal positive cells did not co-express the c-Fos protein (Figure 2, right, yellow arrows).

### Overall expression profile for c-Fos and β- galactosidase

The number of c-Fos and β-gal positive pixels, which includes both histological-positive cell bodies as well as associated cellular processes, paralleled the number of labeled cell bodies. The number of c-Fos labeled pixels in the nTS of MSG animals was significantly greater than the number of labeled pixels in all other groups ([F (1, 3) = 5.64, p<0.05]; Figure 3D). In contrast, the number of β-gal labeled pixels was not different between groups ([F (1, 3) = 1.23, p = 0.36]). Finally, the percentage of nTS that contained labeled β-gal pixels was similar to that observed when the number of β-gal labeled pixels was not normalized to nTS area, with no significant differences between groups at any nTS level ([F (1, 3) = 0.55, p = 0.66]; Figure S39). Together, these results suggest that β-gal staining in the nTS is unaffected by changes in stimulation conditions.

## Discussion

The immediate early gene, c-Fos, is widely used as a marker of brain activity. However, traditional c-Fos expression is confined to the nucleus of activated cells, making it impossible to discern the morphology of activated cells. In this regard, Fos-tau-LacZ transgenic mice make it possible to visualize the entire cellular structure (axons, dendrites and cell bodies) of activated neurons for the first time. Yet, because LacZ/β-gal expression has not been thoroughly characterized previously in brainstem areas, such as the nTS, the present study sought to quantify both c-Fos β-gal enzymatic activity as well as c-Fos protein activity in the nTS under a variety of experimental conditions.

### β - galactosidase activity

The number of β-gal positive cell bodies and the number of β-gal positive pixels (representing cell bodies and associated cytological architecture) were not significantly different across all experimental conditions (Figures 2 and 3). Moreover, the dense network of β-gal staining (ex. Figure 1) made it impossible to discern the morphology of activated cells within the nTS- regardless of experimental condition. This is surprising as β-gal serves as a reliable functional activity marker in other brain areas of FTL mice, including the hippocampus and visual cortex [1], [3]. One explanation for this disparity may be related to the prolonged period of time that the transgene product (β-gal) remains in the cell bodies and neuronal processes in FTL mice (as much as 24–48 hours post stimulus; [3]). In support of this idea, previous reports using FTL mice employed extreme deprivation protocols to limit baseline β-gal activity (e.g. three days in total darkness prior to stimulation; [1]) or examined activity patterns in a region of the brain with much greater area (e.g. hippocampus; [9]) than the nTS, which could be subject to compartmentalized variation in β-gal activity.

Additionally, the nTS processes and integrates multiple types of sensory and motor information [10], and may evince a high basal level of β-gal activity as a result of being responsive to multiple sources of stimulation. However, other brainstem areas adjacent to the nTS, such as the dorsal cochlear nucleus (audition) as well as the reticular formation (sleep-wake cycle), receive near-constant stimulation, but do not show a similar high basal level of β-gal activity (see Figure 1). It may be that the types of information processed within the nTS (e.g. taste, viscerosensory, cardiovascular) are more sensitive to c-Fos activation, and the resulting β-gal enzyme, than nearby brain areas.

Unfortunately, it is impossible to eliminate all sources of external stimuli that are processed within the nTS- especially as some of this information is related to autonomic and homeostatic regulation, including viscerosensory and cardiovascular signals from the gastrointestinal tract and the carotid bodies, respectively [10]. We designed our experimental conditions to limit extraneous stimulation by including a group that received no food or water overnight (i.e. No Food/Water). Importantly, the No Food/Water group most closely mimics the light deprivation experimental procedures previously reported to be optimal for β-gal activity in other studies [1] without causing unnecessary stress to the animals, which itself can influence β-gal activity in FTL mice [9]. Surprisingly, even elimination of this external influence was ineffective in decreasing the basal amount of β-gal staining.

Interestingly, previous research reported increased β-gal activity (as measured by Xgal histochemistry) following CO_2_ exposure in brainstem areas, including the nTS [11]. Although Xgal histochemistry, as used by previous researchers, differs from the β-gal fluorescent immunohistochemistry used in the present study, it is unlikely that differences in histochemical methodology can explain the observed differences in β-gal activity for several reasons. First, other researchers report anatomical congruence between staining by Xgal reaction and β-gal antibody [1], [3]. Second, the β-gal antibody used in the current study was thoroughly tested for specificity in our lab with the appropriate antibody controls, including a lack of staining in LacZ negative animals, and an absence of staining when the primary antibody was omitted. Thus, the reason for this disparity is unknown.

### Fos-LI

As expected, the time course of Fos-LI and number of c-Fos positive pixels (Figures 2 and 3) were in accord with previous studies, which report c-Fos protein expression peaks ∼1 hour post stimulation and returns to basal levels by ∼4 hours [5]. However, unusual punctate, basal c-Fos protein staining was found in all unstimulated groups as well as the MSG 5 h Post group (Figure 2), and high c-Fos background label (i.e. non-specific immunofluorescence) was present in tissue sections from MSG- stimulated FTL mice (but was not present in WT control mice using the same reagents; Figure S38). Furthermore, in the MSG group, Fos-LI was found in the cellular processes of some c-Fos positive cells, in addition to stained cell bodies (Figure 2 inset). These protein staining patterns are unusual as the punctate staining is much smaller in size than normal c-Fos positive cells and c-Fos protein is normally localized to the nucleus of active cells (Figure 2, red vs. blue arrows). The cause of this atypical staining may be related to the tau transgene present in FTL animals, which normally shuttles β-gal throughout the cytoplasm of activated cells, but may also have a secondary consequence that results from transporting c-Fos protein from outside the nucleus of cells [12].

Finally, the number of c-Fos/β-gal double labeled cells paralleled the results of Fos-LI alone. The number of double cell bodies was greatest in the MSG group; whereas, few double labeled cell bodies were found in all other groups (Figure 3C). However, not all c-Fos positive cells co-localized with β-gal (Figure 2, yellow arrows), suggesting that the unchanged level of β-gal staining observed across all experimental groups does not include all cells within the nTS that express c-Fos. This is further supported by the lack of change in β-gal staining between the MSG group and the MSG 5 h Post group- a time point at which β-gal is normally at its greatest level of expression post stimulation [4]. Together, this suggests that β-gal staining in the nTS represents a stable population of β-gal expressing cells that is not affected by environmental of stimulation conditions.

### c-Fos activity within the nTS

Although the goal of this study was to characterize c-Fos expression in the nTS in response MSG taste stimulation, it is important to note that the nTS serves as a convergence site for multiples types of sensory and motor information. Thus, c-Fos label within the nTS may result not only from MSG taste stimulation, but also from other sources of input as well. For instance, caudal portions of the nTS receive both viscerosensory and cardiovascular information from branches of the vagus and glossopharyngeal nerves [13], [14]; whereas the rostral nTS receives both orosensory and taste input [15], [16]. Thus, Fos-LI observed within the nTS may result from a combination of taste and indirect sources of activation such our stimulation procedures (i.e. rehydration, post-oral detection of MSG, oromotor behavior, etc.).

### Utility of FTL mice

We observed high basal β-gal reactivity in the nTS of FTL mice, which suggests that this transgenic mouse line would likely complicate experiments focused on connectivity and morphology within the nTS. Furthermore, our results suggest that other brain areas may also show a similar high level of β-gal activity. Unfortunately, it is impossible to predict which brain areas may show increased tonic β-gal staining. Areas adjacent to the nTS, such as the dorsal cochlear nucleus and reticular formation, presumably experience similar levels of increased neuronal activity as the nTS. Yet, β-gal staining in both of these brain areas is greatly reduced compared to that observed in the nTS; whereas, β-gal staining is very dense in the hypoglossal nucleus (involved in tongue movements and swallowing).

Though FTL mice are not suitable for work in the nTS, other researchers use FTL mice to explore the connections between activated neurons other brain areas, including both the visual cortex as well as the hippocampus However, one feature of FTL mice yet to be utilized is the prolonged perdurance of β-gal activity in FTL mice, which is much longer than both traditional c-Fos protein and c-Fos mRNA activity. β-gal can be seen 24–48 hours post stimulation [3]; whereas c-Fos protein lasts 1–3 hours and c-Fos mRNA peaks 30 minutes after stimulation [5]. This prolonged labeling of c-Fos activated cells by β-gal enables- in conjunction with the rapid induction of c-Fos mRNA- measurement of c-Fos activity in response to two stimuli, separated temporally, within the same animal (e.g. β-galactosidase expression and c-Fos mRNA expression).

## Supporting Information

Figure S1 **Caudal Level of the nTS of FTL Mouse that Received No Stimulation and No Food or Water Overnight.** Red is c-Fos protein, green is β-gal staining, blue is a Nissl counterstain.
(TIF)

Figure S2 **Intermediate Level of the nTS of FTL Mouse that Received No Stimulation and No Food or Water Overnight.** Red is c-Fos protein, green is β-gal staining, blue is a Nissl counterstain.
(TIF)

Figure S3 **Rostral Level of the nTS of FTL Mouse that Received No Stimulation and No Food or Water Overnight.** Red is c-Fos protein, green is β-gal staining, blue is a Nissl counterstain.
(TIF)

Figure S4 **Caudal Level of the nTS of FTL Mouse that Received No Stimulation.** Red is c-Fos protein, green is β-gal staining, blue is a Nissl counterstain.
(TIF)

Figure S5 **Intermediate Level of the nTS of FTL Mouse that Received No Stimulation.** Red is c-Fos protein, green is β-gal staining, blue is a Nissl counterstain.
(TIF)

Figure S6 **Rostral Level of the nTS of FTL Mouse that Received No Stimulation.** Red is c-Fos protein, green is β-gal staining, blue is a Nissl counterstain.
(TIF)

Figure S7 **Caudal Level of the nTS of FTL Mouse that Received No Stimulation and No Food or Water Overnight.** Red is c-Fos protein, green is β-gal staining, blue is a Nissl counterstain.
(TIF)

Figure S8 **Intermediate Level of the nTS of FTL Mouse that Received No Stimulation and No Food or Water Overnight.** Red is c-Fos protein, green is β-gal staining, blue is a Nissl counterstain.
(TIF)

Figure S9 **Rostral Level of the nTS of FTL Mouse that Received No Stimulation and No Food or Water Overnight.** Red is c-Fos protein, green is β-gal staining, blue is a Nissl counterstain.
(TIF)

Figure S10 **Caudal Level of the nTS of FTL Mouse that Received No Stimulation.** Red is c-Fos protein, green is β-gal staining, blue is a Nissl counterstain.
(TIF)

Figure S11 **Intermediate Level of the nTS of FTL Mouse that Received No Stimulation.** Red is c-Fos protein, green is β-gal staining, blue is a Nissl counterstain.
(TIF)

Figure S12 **Rostral Level of the nTS of FTL Mouse that Received No Stimulation.** Red is c-Fos protein, green is β-gal staining, blue is a Nissl counterstain.
(TIF)

Figure S13 **Caudal Level of the nTS of FTL Mouse stimulated with 150 mM MSG and perfused 45 min post stimulation.** Red is c-Fos protein, green is β-gal staining, blue is a Nissl counterstain.
(TIF)

Figure S14 **Intermediate Level of the nTS of FTL Mouse stimulated with 150 mM MSG and perfused 45 min post stimulation.** Red is c-Fos protein, green is β-gal staining, blue is a Nissl counterstain.
(TIF)

Figure S15 **Rostral Level of the nTS of FTL Mouse stimulated with 150 mM MSG and perfused 45 min post stimulation.** Red is c-Fos protein, green is β-gal staining, blue is a Nissl counterstain.
(TIF)

Figure S16 **Caudal Level of the nTS of FTL Mouse stimulated with 150 mM MSG and perfused 45 min post stimulation.** Red is c-Fos protein, green is β-gal staining, blue is a Nissl counterstain.
(TIF)

Figure S17 **Intermediate Level of the nTS of FTL Mouse stimulated with 150 mM MSG and perfused 45 min post stimulation.** Red is c-Fos protein, green is β-gal staining, blue is a Nissl counterstain.
(TIF)

Figure S18 **Rostral Level of the nTS of FTL Mouse stimulated with 150 mM MSG and perfused 45 min post stimulation.** Red is c-Fos protein, green is β-gal staining, blue is a Nissl counterstain.
(TIF)

Figure S19 **Caudal Level of the nTS of FTL Mouse that Received No Stimulation and No Food or Water Overnight.** Red is c-Fos protein, green is β-gal staining, blue is a Nissl counterstain.
(TIF)

Figure S20 **Intermediate Level of the nTS of FTL Mouse that Received No Stimulation and No Food or Water Overnight.** Red is c-Fos protein, green is β-gal staining, blue is a Nissl counterstain.
(TIF)

Figure S21 **Rostral Level of the nTS of FTL Mouse that Received No Stimulation and No Food or Water Overnight.** Red is c-Fos protein, green is β-gal staining, blue is a Nissl counterstain.
(TIF)

Figure S22 **Caudal Level of the nTS of FTL Mouse that Received No Stimulation.** Red is c-Fos protein, green is β-gal staining, blue is a Nissl counterstain.
(TIF)

Figure S23 **Intermediate Level of the nTS of FTL Mouse that Received No Stimulation.** Red is c-Fos protein, green is β-gal staining, blue is a Nissl counterstain.
(TIF)

Figure S24 **Rostral Level of the nTS of FTL Mouse that Received No Stimulation.** Red is c-Fos protein, green is β-gal staining, blue is a Nissl counterstain.
(TIF)

Figure S25 **Caudal Level of the nTS of FTL Mouse stimulated with 150 mM MSG and perfused 5 hr post stimulation.** Red is c-Fos protein, green is β-gal staining, blue is a Nissl counterstain.
(TIF)

Figure S26 **Intermediate Level of the nTS of FTL Mouse stimulated with 150 mM MSG and perfused 5 hr post stimulation.** Red is c-Fos protein, green is β-gal staining, blue is a Nissl counterstain.
(TIF)

Figure S27 **Rostral Level of the nTS of FTL Mouse stimulated with 150 mM MSG and perfused 5 hr post stimulation.** Red is c-Fos protein, green is β-gal staining, blue is a Nissl counterstain.
(TIF)

Figure S28 **Caudal Level of the nTS of FTL Mouse stimulated with 150 mM MSG and perfused 5 hr post stimulation.** Red is c-Fos protein, green is β-gal staining, blue is a Nissl counterstain.
(TIF)

Figure S29 **Intermediate Level of the nTS of FTL Mouse stimulated with 150 mM MSG and perfused 5 hr post stimulation.** Red is c-Fos protein, green is β-gal staining, blue is a Nissl counterstain.
(TIF)

Figure S30 **Rostral Level of the nTS of FTL Mouse stimulated with 150 mM MSG and perfused 5 hr post stimulation.** Red is c-Fos protein, green is β-gal staining, blue is a Nissl counterstain.
(TIF)

Figure S31 **Caudal Level of the nTS of FTL Mouse stimulated with 150 mM MSG and perfused 5 hr post stimulation.** Red is c-Fos protein, green is β-gal staining, blue is a Nissl counterstain.
(TIF)

Figure S32 **Intermediate Level of the nTS of FTL Mouse stimulated with 150 mM MSG and perfused 5 hr post stimulation.** Red is c-Fos protein, green is β-gal staining, blue is a Nissl counterstain.
(TIF)

Figure S33 **Rostral Level of the nTS of FTL Mouse stimulated with 150 mM MSG and perfused 5 hr post stimulation.** Red is c-Fos protein, green is β-gal staining, blue is a Nissl counterstain.
(TIF)

Figure S34 **Caudal Level of the nTS of FTL Mouse stimulated with 150 mM MSG and perfused 45 min post stimulation.** Red is c-Fos protein, green is β-gal staining, blue is a Nissl counterstain.
(TIF)

Figure S35 **Intermediate Level of the nTS of FTL Mouse stimulated with 150 mM MSG and perfused 45 min post stimulation.** Red is c-Fos protein, green is β-gal staining, blue is a Nissl counterstain.
(TIF)

Figure S36 **Rostral Level of the nTS of FTL Mouse stimulated with 150 mM MSG and perfused 45 min post stimulation.** Red is c-Fos protein, green is β-gal staining, blue is a Nissl counterstain.
(TIF)

Figure S37 **β – galactosidase staining is found in the olfactory bulb of FTL mice, with particular concentration in the glomerular and mitral cell layers.** **Left**: Atlas image of olfactory bulb. AOB: accessory olfactory bulb, GrA: granule cell layer of the accessory olfactory bulb, EPI: external plexiform layer, GrO: granular cell layer of the olfactory bulb, Mi: mitral cell layer of the olfactory bulb, Gl: glomerular layer of the olfactory bulb, IPI: internal plexiform layer of the olfactory bulb, AOA: anterior olfactory area. Images modified from Paxinos The Mouse Brain in Stereotaxic Coordinates, 2nd Edition. **Middle** and **Right**: Photomicrographs of and c-Fos protein (**Middle**) and fluorescent β – galactosidase (**Right**) staining in the brainstem of a FTL mouse that received no stimulation (Unstim). Images converted to greyscale colors for clarity. An enlargement of each stain is presented as an inset to the right of each image.
(TIF)

Figure S38 **High background Fos-LI staining is present in the nTS of FTL, but not WT, mice stimulated with MSG.** Photomicrographs of Fos-LI in the Rostral level of the nTS of a wild type (WT; **Left**) and FTL (**Right**) mouse. Both animals were stimulated with 150 mM MSG in the exact same way and brain tissue from each animal was processed for Fos-LI using the same reagents. Images converted to greyscale colors for clarity. Background staining is defined as ‘non-specific immunofluorescence that was not localized to cell nuclei.’
(TIF)

Figure S39 **β – galactosidase staining is similar across all experimental conditions even when normalized to nTS size.** The percent of nTS labeled with β-gal (calculated by dividing the number of β-gal labeled pixels that exceeded threshold by the total number of pixels within the nTS) was not statistically different between groups (p = 0.66).
(TIF)
