# Supplementary figures and images for: Beta-Galactosidase Staining in the Nucleus of the Solitary Tract of Fos-Tau-LacZ Mice Is Unaffected by Monosodium Glutamate Taste Stimulation
Source: PLoS One. 2014 Sep 5;9(9):e107238. doi: 10.1371/journal.pone.0107238 (PMC4156431; doi:10.1371/journal.pone.0107238)

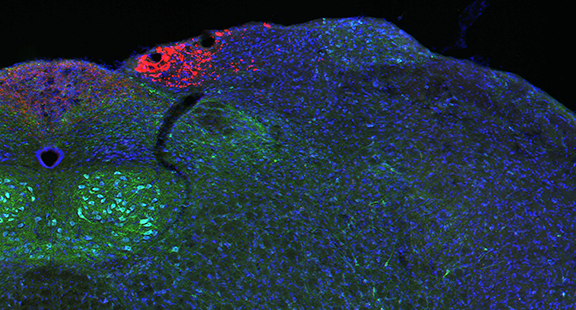

Supplement: Figure S1 — Caudal Level of the nTS of FTL Mouse that Received No Stimulation and No Food or Water Overnight. Red is c-Fos protein, green is β-gal staining, blue is a Nissl counterstain. (TIF) [file pone.0107238.s001.tif]

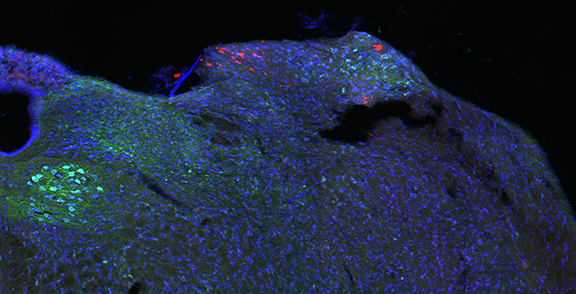

Supplement: Figure S2 — Intermediate Level of the nTS of FTL Mouse that Received No Stimulation and No Food or Water Overnight. Red is c-Fos protein, green is β-gal staining, blue is a Nissl counterstain. (TIF) [file pone.0107238.s002.tif]

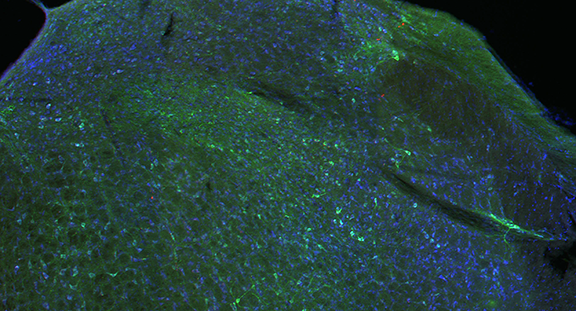

Supplement: Figure S3 — Rostral Level of the nTS of FTL Mouse that Received No Stimulation and No Food or Water Overnight. Red is c-Fos protein, green is β-gal staining, blue is a Nissl counterstain. (TIF) [file pone.0107238.s003.tif]

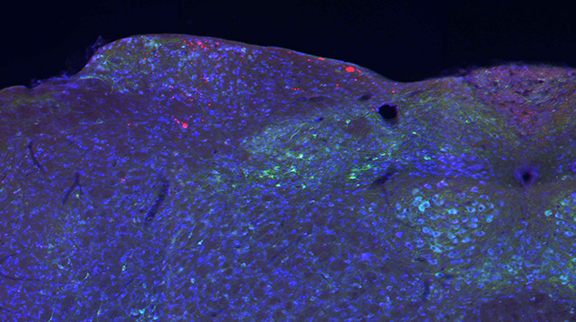

Supplement: Figure S4 — Caudal Level of the nTS of FTL Mouse that Received No Stimulation. Red is c-Fos protein, green is β-gal staining, blue is a Nissl counterstain. (TIF) [file pone.0107238.s004.tif]

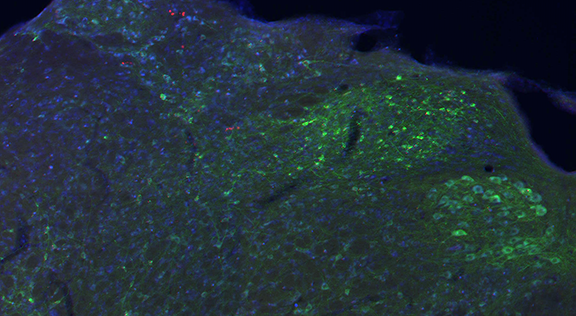

Supplement: Figure S5 — Intermediate Level of the nTS of FTL Mouse that Received No Stimulation. Red is c-Fos protein, green is β-gal staining, blue is a Nissl counterstain. (TIF) [file pone.0107238.s005.tif]

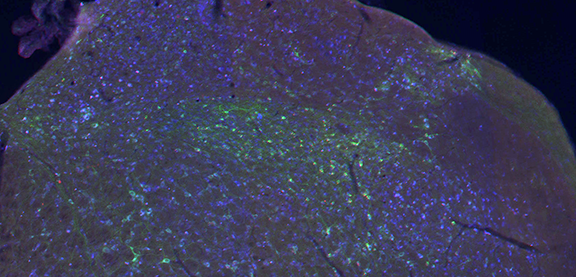

Supplement: Figure S6 — Rostral Level of the nTS of FTL Mouse that Received No Stimulation. Red is c-Fos protein, green is β-gal staining, blue is a Nissl counterstain. (TIF) [file pone.0107238.s006.tif]

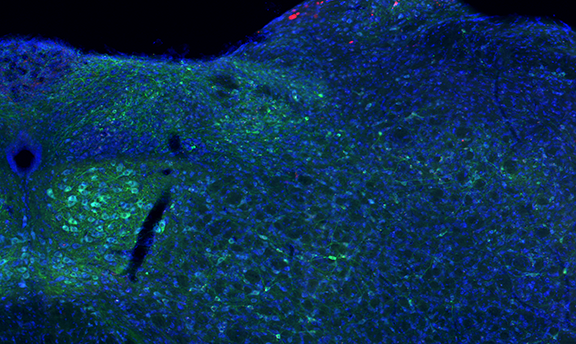

Supplement: Figure S7 — Caudal Level of the nTS of FTL Mouse that Received No Stimulation and No Food or Water Overnight. Red is c-Fos protein, green is β-gal staining, blue is a Nissl counterstain. (TIF) [file pone.0107238.s007.tif]

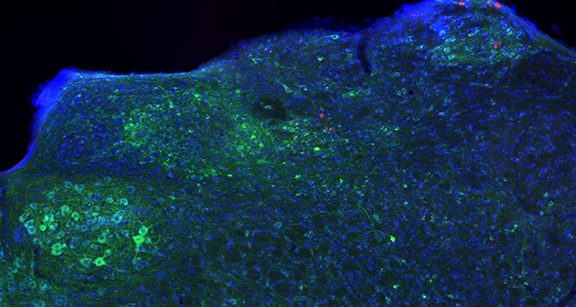

Supplement: Figure S8 — Intermediate Level of the nTS of FTL Mouse that Received No Stimulation and No Food or Water Overnight. Red is c-Fos protein, green is β-gal staining, blue is a Nissl counterstain. (TIF) [file pone.0107238.s008.tif]

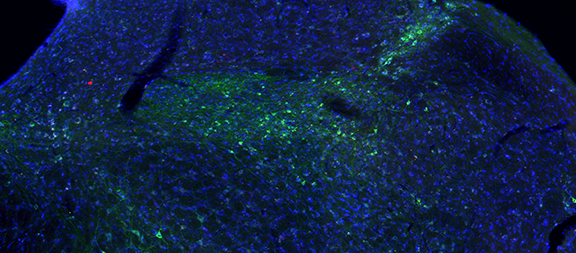

Supplement: Figure S9 — Rostral Level of the nTS of FTL Mouse that Received No Stimulation and No Food or Water Overnight. Red is c-Fos protein, green is β-gal staining, blue is a Nissl counterstain. (TIF) [file pone.0107238.s009.tif]

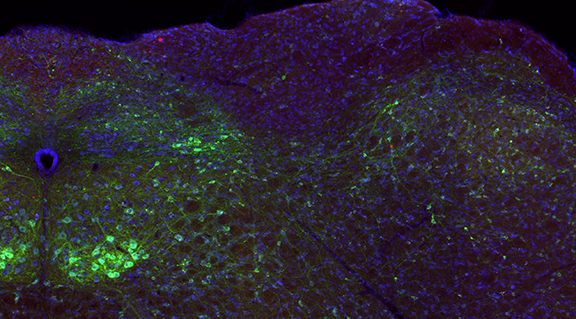

Supplement: Figure S10 — Caudal Level of the nTS of FTL Mouse that Received No Stimulation. Red is c-Fos protein, green is β-gal staining, blue is a Nissl counterstain. (TIF) [file pone.0107238.s010.tif]

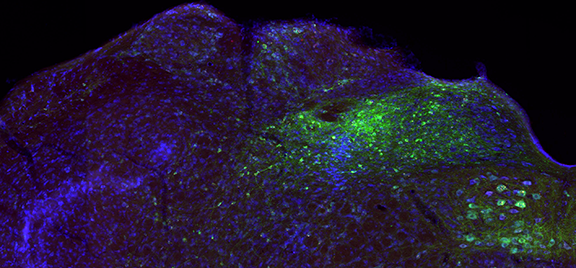

Supplement: Figure S11 — Intermediate Level of the nTS of FTL Mouse that Received No Stimulation. Red is c-Fos protein, green is β-gal staining, blue is a Nissl counterstain. (TIF) [file pone.0107238.s011.tif]

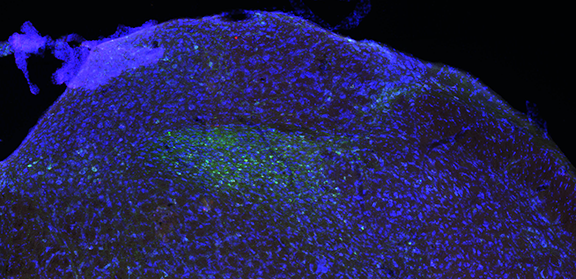

Supplement: Figure S12 — Rostral Level of the nTS of FTL Mouse that Received No Stimulation. Red is c-Fos protein, green is β-gal staining, blue is a Nissl counterstain. (TIF) [file pone.0107238.s012.tif]

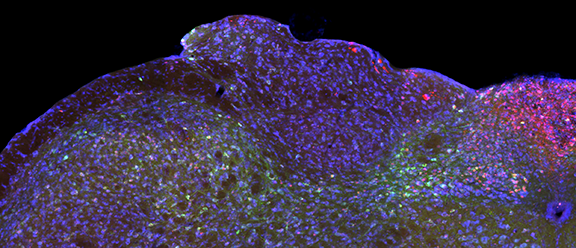

Supplement: Figure S13 — Caudal Level of the nTS of FTL Mouse stimulated with 150 mM MSG and perfused 45 min post stimulation. Red is c-Fos protein, green is β-gal staining, blue is a Nissl counterstain. (TIF) [file pone.0107238.s013.tif]

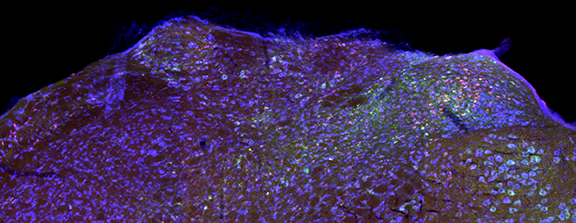

Supplement: Figure S14 — Intermediate Level of the nTS of FTL Mouse stimulated with 150 mM MSG and perfused 45 min post stimulation. Red is c-Fos protein, green is β-gal staining, blue is a Nissl counterstain. (TIF) [file pone.0107238.s014.tif]

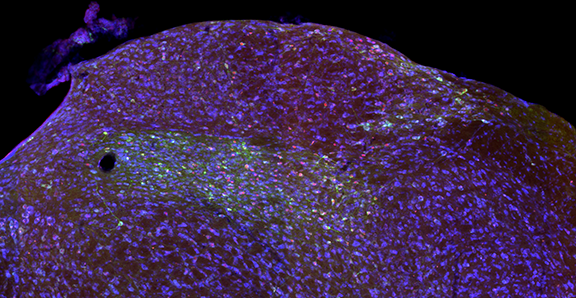

Supplement: Figure S15 — Rostral Level of the nTS of FTL Mouse stimulated with 150 mM MSG and perfused 45 min post stimulation. Red is c-Fos protein, green is β-gal staining, blue is a Nissl counterstain. (TIF) [file pone.0107238.s015.tif]

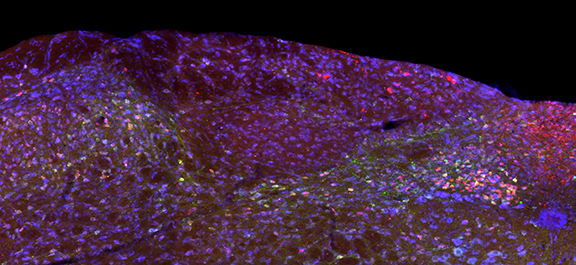

Supplement: Figure S16 — Caudal Level of the nTS of FTL Mouse stimulated with 150 mM MSG and perfused 45 min post stimulation. Red is c-Fos protein, green is β-gal staining, blue is a Nissl counterstain. (TIF) [file pone.0107238.s016.tif]

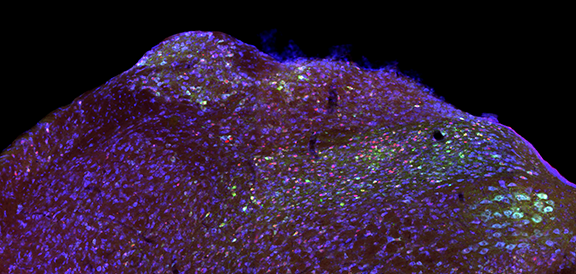

Supplement: Figure S17 — Intermediate Level of the nTS of FTL Mouse stimulated with 150 mM MSG and perfused 45 min post stimulation. Red is c-Fos protein, green is β-gal staining, blue is a Nissl counterstain. (TIF) [file pone.0107238.s017.tif]

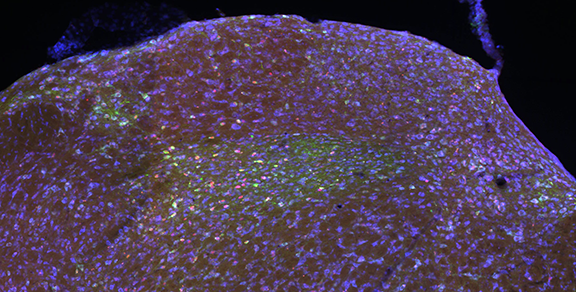

Supplement: Figure S18 — Rostral Level of the nTS of FTL Mouse stimulated with 150 mM MSG and perfused 45 min post stimulation. Red is c-Fos protein, green is β-gal staining, blue is a Nissl counterstain. (TIF) [file pone.0107238.s018.tif]

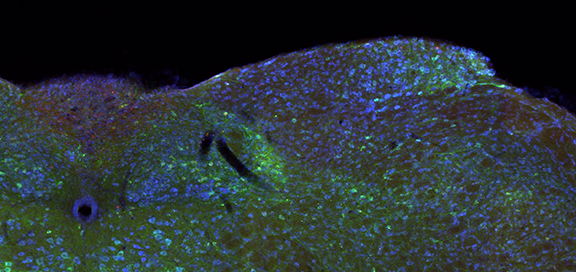

Supplement: Figure S19 — Caudal Level of the nTS of FTL Mouse that Received No Stimulation and No Food or Water Overnight. Red is c-Fos protein, green is β-gal staining, blue is a Nissl counterstain. (TIF) [file pone.0107238.s019.tif]

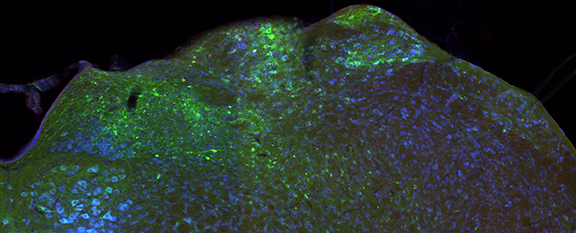

Supplement: Figure S20 — Intermediate Level of the nTS of FTL Mouse that Received No Stimulation and No Food or Water Overnight. Red is c-Fos protein, green is β-gal staining, blue is a Nissl counterstain. (TIF) [file pone.0107238.s020.tif]

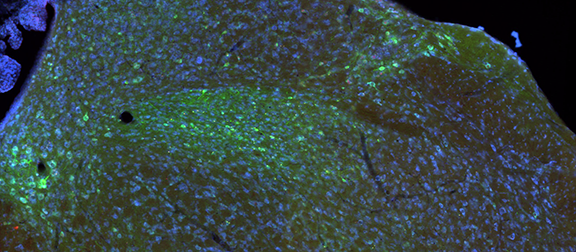

Supplement: Figure S21 — Rostral Level of the nTS of FTL Mouse that Received No Stimulation and No Food or Water Overnight. Red is c-Fos protein, green is β-gal staining, blue is a Nissl counterstain. (TIF) [file pone.0107238.s021.tif]

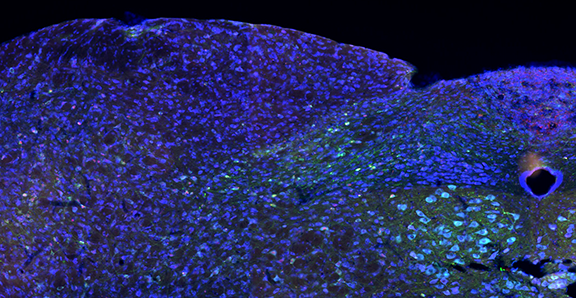

Supplement: Figure S22 — Caudal Level of the nTS of FTL Mouse that Received No Stimulation. Red is c-Fos protein, green is β-gal staining, blue is a Nissl counterstain. (TIF) [file pone.0107238.s022.tif]

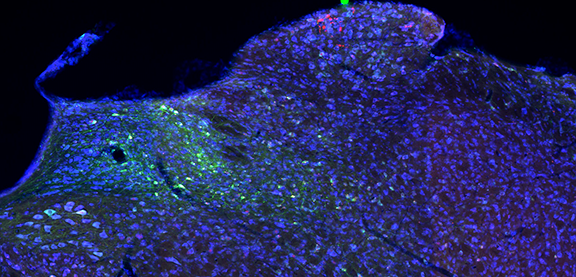

Supplement: Figure S23 — Intermediate Level of the nTS of FTL Mouse that Received No Stimulation. Red is c-Fos protein, green is β-gal staining, blue is a Nissl counterstain. (TIF) [file pone.0107238.s023.tif]

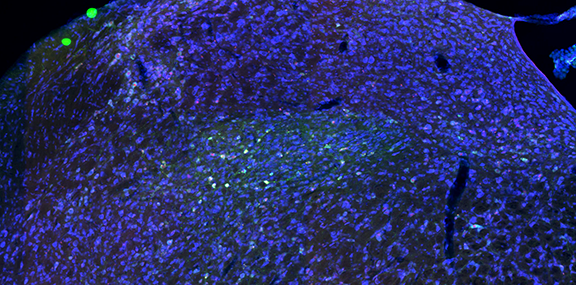

Supplement: Figure S24 — Rostral Level of the nTS of FTL Mouse that Received No Stimulation. Red is c-Fos protein, green is β-gal staining, blue is a Nissl counterstain. (TIF) [file pone.0107238.s024.tif]

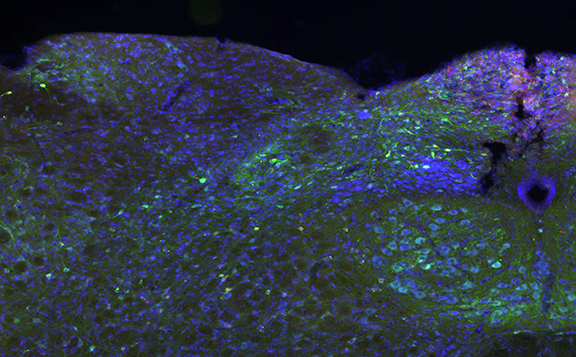

Supplement: Figure S25 — Caudal Level of the nTS of FTL Mouse stimulated with 150 mM MSG and perfused 5 hr post stimulation. Red is c-Fos protein, green is β-gal staining, blue is a Nissl counterstain. (TIF) [file pone.0107238.s025.tif]

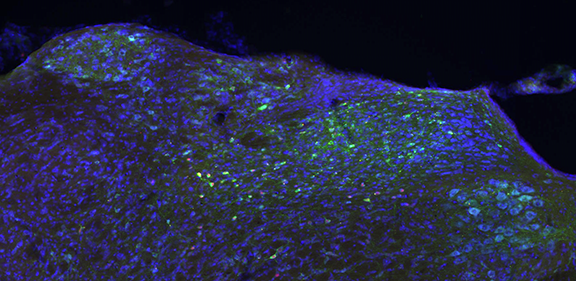

Supplement: Figure S26 — Intermediate Level of the nTS of FTL Mouse stimulated with 150 mM MSG and perfused 5 hr post stimulation. Red is c-Fos protein, green is β-gal staining, blue is a Nissl counterstain. (TIF) [file pone.0107238.s026.tif]

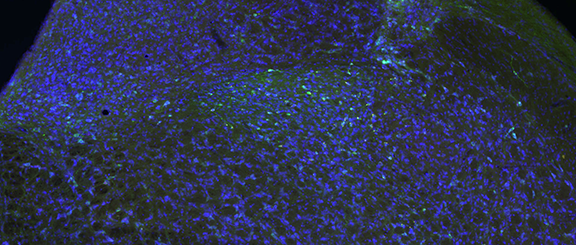

Supplement: Figure S27 — Rostral Level of the nTS of FTL Mouse stimulated with 150 mM MSG and perfused 5 hr post stimulation. Red is c-Fos protein, green is β-gal staining, blue is a Nissl counterstain. (TIF) [file pone.0107238.s027.tif]

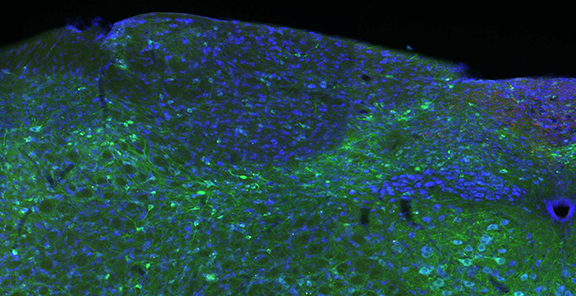

Supplement: Figure S28 — Caudal Level of the nTS of FTL Mouse stimulated with 150 mM MSG and perfused 5 hr post stimulation. Red is c-Fos protein, green is β-gal staining, blue is a Nissl counterstain. (TIF) [file pone.0107238.s028.tif]

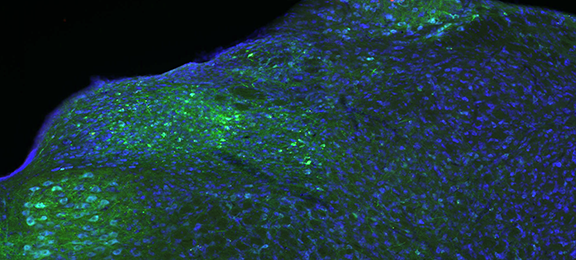

Supplement: Figure S29 — Intermediate Level of the nTS of FTL Mouse stimulated with 150 mM MSG and perfused 5 hr post stimulation. Red is c-Fos protein, green is β-gal staining, blue is a Nissl counterstain. (TIF) [file pone.0107238.s029.tif]

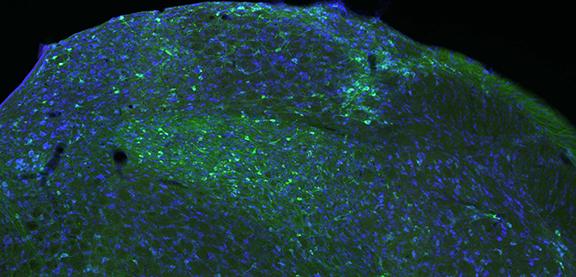

Supplement: Figure S30 — Rostral Level of the nTS of FTL Mouse stimulated with 150 mM MSG and perfused 5 hr post stimulation. Red is c-Fos protein, green is β-gal staining, blue is a Nissl counterstain. (TIF) [file pone.0107238.s030.tif]

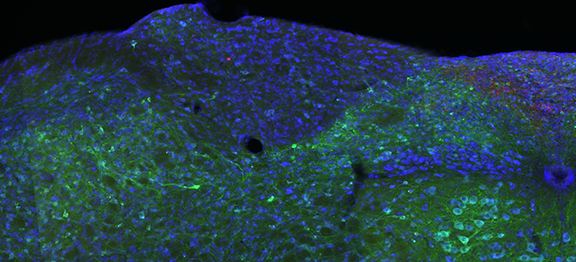

Supplement: Figure S31 — Caudal Level of the nTS of FTL Mouse stimulated with 150 mM MSG and perfused 5 hr post stimulation. Red is c-Fos protein, green is β-gal staining, blue is a Nissl counterstain. (TIF) [file pone.0107238.s031.tif]

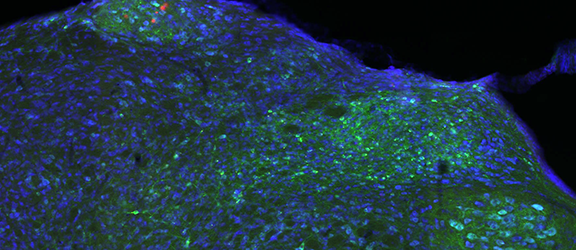

Supplement: Figure S32 — Intermediate Level of the nTS of FTL Mouse stimulated with 150 mM MSG and perfused 5 hr post stimulation. Red is c-Fos protein, green is β-gal staining, blue is a Nissl counterstain. (TIF) [file pone.0107238.s032.tif]

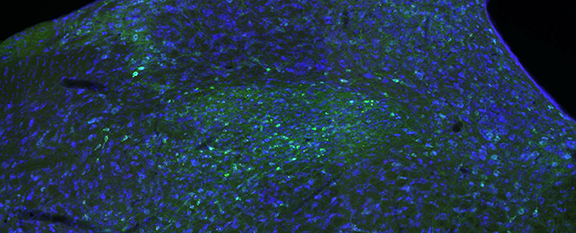

Supplement: Figure S33 — Rostral Level of the nTS of FTL Mouse stimulated with 150 mM MSG and perfused 5 hr post stimulation. Red is c-Fos protein, green is β-gal staining, blue is a Nissl counterstain. (TIF) [file pone.0107238.s033.tif]

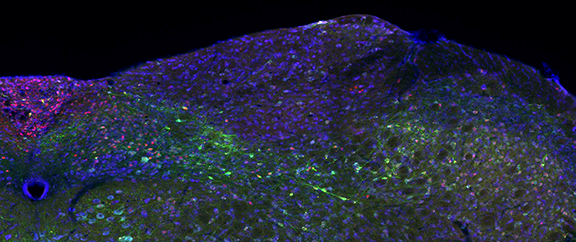

Supplement: Figure S34 — Caudal Level of the nTS of FTL Mouse stimulated with 150 mM MSG and perfused 45 min post stimulation. Red is c-Fos protein, green is β-gal staining, blue is a Nissl counterstain. (TIF) [file pone.0107238.s034.tif]

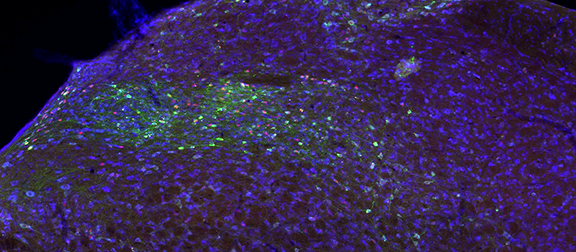

Supplement: Figure S35 — Intermediate Level of the nTS of FTL Mouse stimulated with 150 mM MSG and perfused 45 min post stimulation. Red is c-Fos protein, green is β-gal staining, blue is a Nissl counterstain. (TIF) [file pone.0107238.s035.tif]

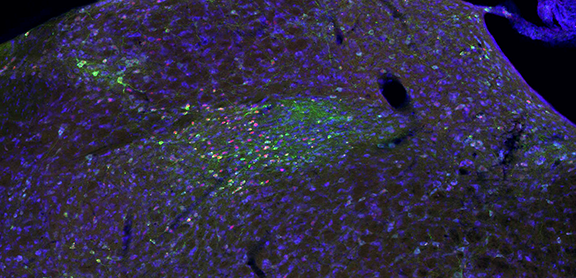

Supplement: Figure S36 — Rostral Level of the nTS of FTL Mouse stimulated with 150 mM MSG and perfused 45 min post stimulation. Red is c-Fos protein, green is β-gal staining, blue is a Nissl counterstain. (TIF) [file pone.0107238.s036.tif]

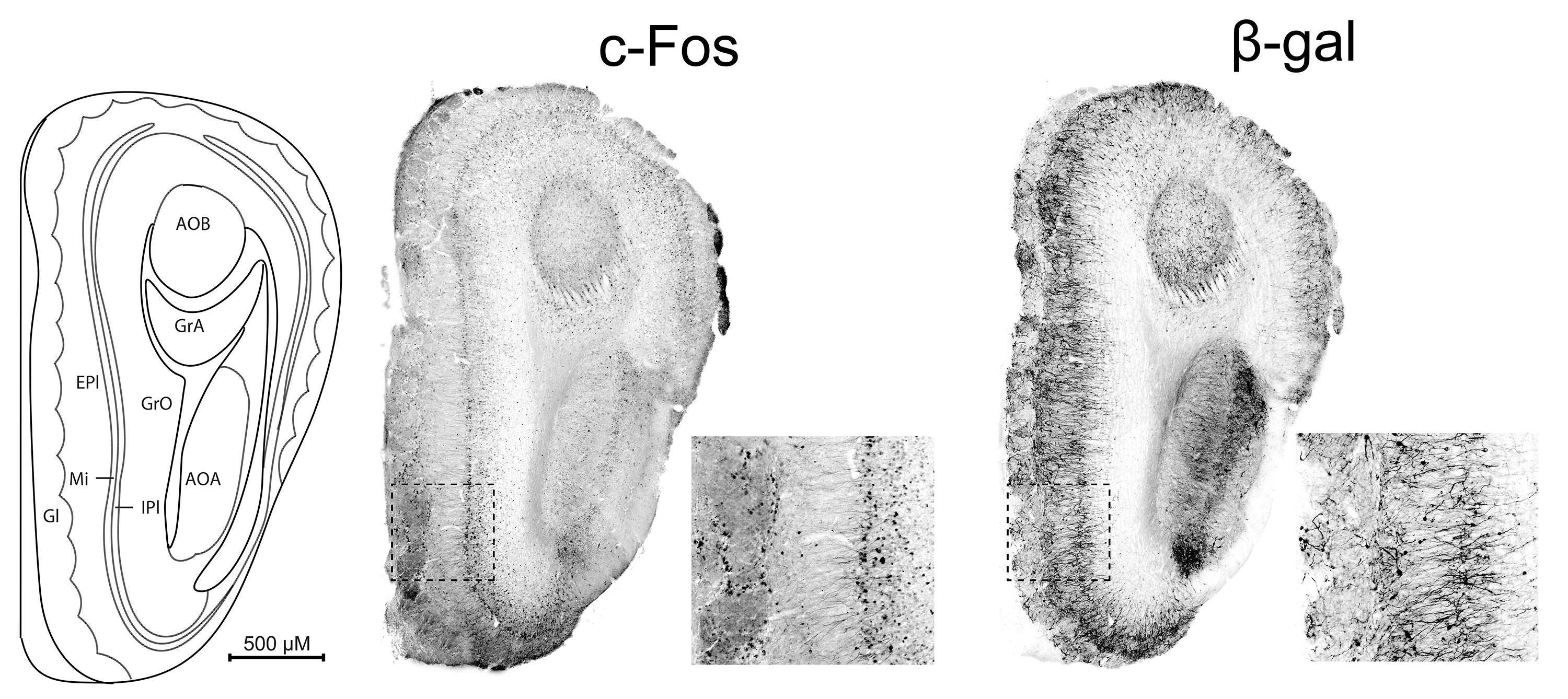

Supplement: Figure S37 — β – galactosidase staining is found in the olfactory bulb of FTL mice, with particular concentration in the glomerular and mitral cell layers. Left: Atlas image of olfactory bulb. AOB: accessory olfactory bulb, GrA: granule cell layer of the accessory olfactory bulb, EPI: external plexiform layer, GrO: granular cell layer of the olfactory bulb, Mi: mitral cell layer of the olfactory bulb, Gl: glomerular layer of the olfactory bulb, IPI: internal plexiform layer of the olfactory bulb, AOA: anterior olfactory area. Images modified from Paxinos The Mouse Brain in Stereotaxic Coordinates, 2nd Edition. Middle and Right: Photomicrographs of and c-Fos protein (Middle) and fluorescent β – galactosidase (Right) staining in the brainstem of a FTL mouse that received no stimulation (Unstim). Images converted to greyscale colors for clarity. An enlargement of each stain is presented as an inset to the right of each image. (TIF) [file pone.0107238.s037.tif]

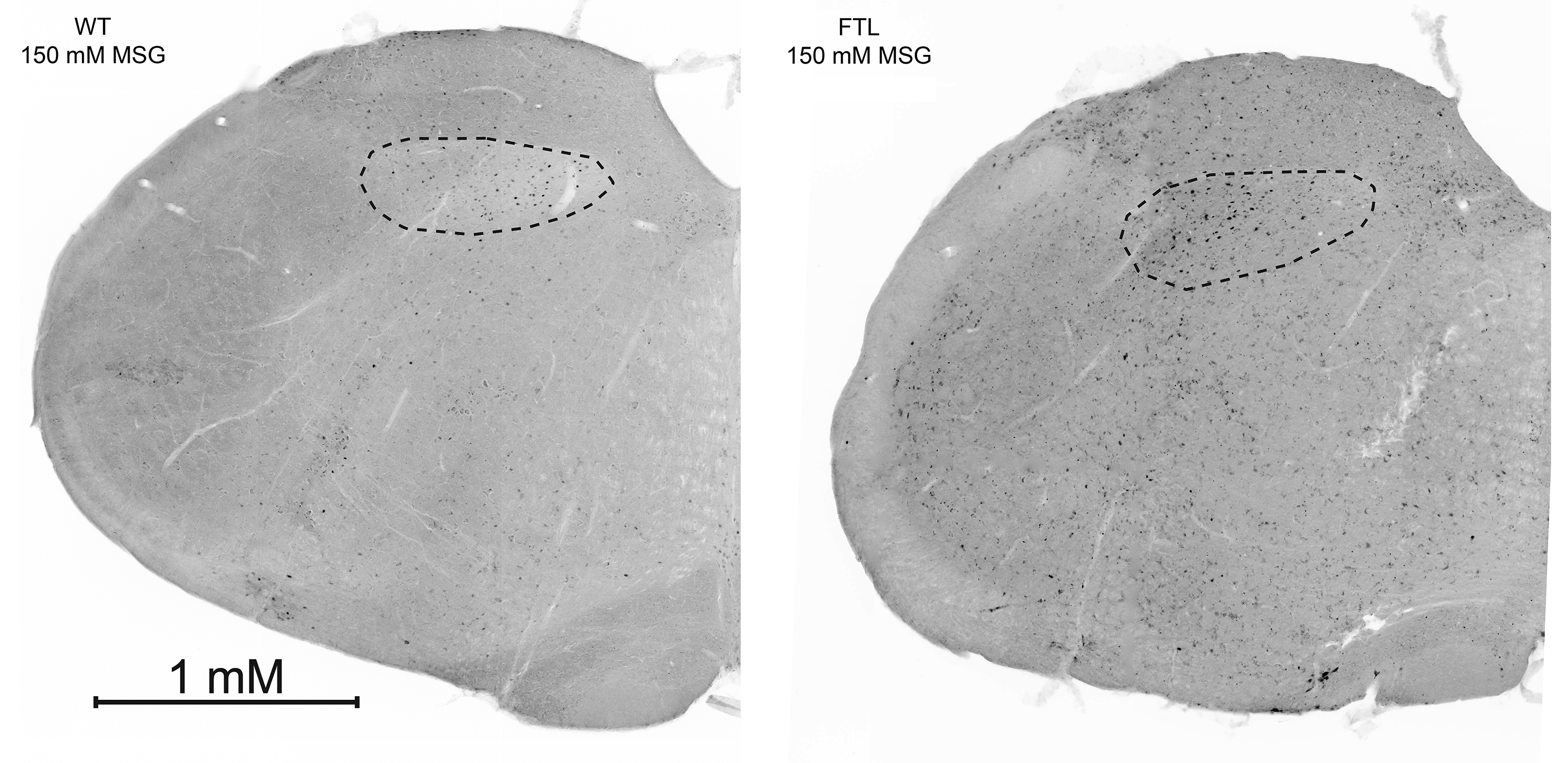

Supplement: Figure S38 — High background Fos-LI staining is present in the nTS of FTL, but not WT, mice stimulated with MSG. Photomicrographs of Fos-LI in the Rostral level of the nTS of a wild type (WT; Left) and FTL (Right) mouse. Both animals were stimulated with 150 mM MSG in the exact same way and brain tissue from each animal was processed for Fos-LI using the same reagents. Images converted to greyscale colors for clarity. Background staining is defined as ‘non-specific immunofluorescence that was not localized to cell nuclei.’ (TIF) [file pone.0107238.s038.tif]

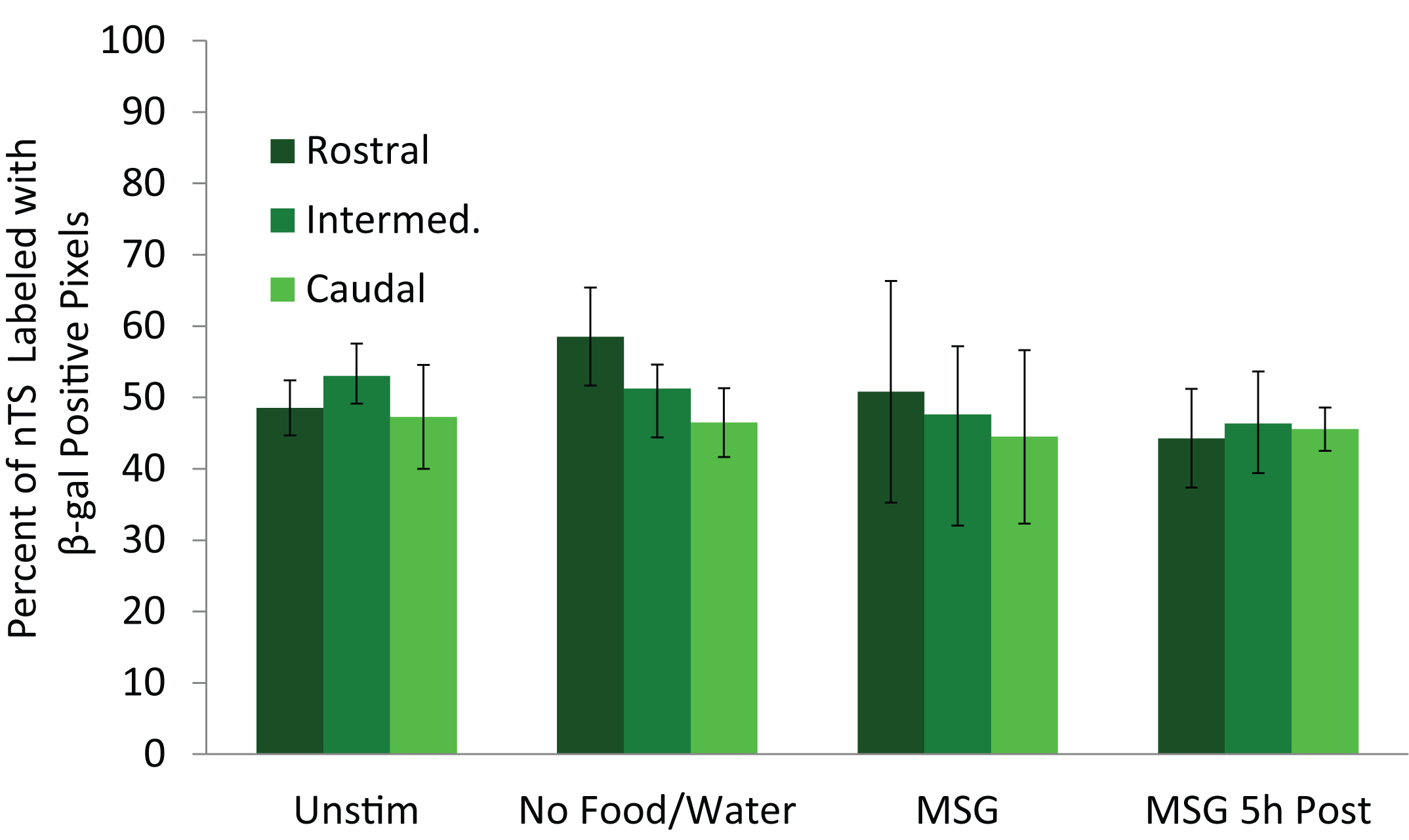

Supplement: Figure S39 — β – galactosidase staining is similar across all experimental conditions even when normalized to nTS size. The percent of nTS labeled with β-gal (calculated by dividing the number of β-gal labeled pixels that exceeded threshold by the total number of pixels within the nTS) was not statistically different between groups (p = 0.66). (TIF) [file pone.0107238.s039.tif]
